# Reply to: fMRI replicability depends upon sufficient individual-level data

**DOI:** 10.1038/s42003-019-0379-5

**Published:** 2019-04-12

**Authors:** Benjamin O. Turner, Tyler Santander, Erick J. Paul, Aron K. Barbey, Michael B. Miller

**Affiliations:** 1 Wee Kim Wee School of Communication and Information, Nanyang Technological University, Singapore, Singapore; 2 Department of Psychological & Brain Sciences, University of California, Santa Barbara, CA USA; 3 Microsoft Corporation, 1 Microsoft Way, Redmond, 98052 WA USA; 4 Beckman Institute for Advanced Science & Technology, University of Illinois at Urbana-Champaign, Urbana, IL USA

**Replying to** D.E. Nee *Communications Biology* 10.1038/s42003-019-0378-6 (2019)

It is well known that the degree to which research replicates depends upon many factors, including sample size and the amount of data per participant. However, previously, little was known as to what constitutes “enough” for either of these factors when it comes to task-based fMRI. Our earlier publication suggested that typical sample sizes may be associated with fairly low replicability. The present exchange further clarifies this finding by highlighting the role of individual-level sampling. Here, we argue that neither sample size nor individual-level sampling alone is sufficient to predict replicability, and that a more productive path forward for the field is to begin to compute these measures as a matter of course, so that the field can gain a better understanding of all of the many drivers of replicability.

In the wake of the reproducibility crisis in Psychology, scientists have increasingly examined the most widely used methods in Cognitive Neuroscience and investigated whether task-based fMRI findings can be replicated. In our recent study^1^, we demonstrated the degree to which group-average fMRI results are surprisingly irreplicable even at sample sizes much larger than typical in the field. The Matters Arising by Nee^2^ in response to our publication argued that this analysis overlooked a critical variable—namely, the amount of per-participant data—which not only also drives replicability, but which was critically low in the examined datasets, limiting the generalizability of the reported findings. We welcome this commentary and believe that constructive dialogue about these important issues will be necessary to advance our understanding of the drivers of replicability in task-based fMRI. Our perspective is that field-wide collaboration—representing a much larger sample of task-based fMRI paradigms and parameters than reported in our prior study and in Nee’s commentary—will ultimately be necessary to make functional neuroimaging a more replicable science: sample size, design efficiency, and individual-level sampling are only several components of the massive parameter space fMRI researchers must navigate when designing studies, acquiring data, constructing models, and preprocessing and analyzing data, and the contribution of many of these components to replicability are still unknown.

We suggest that Nee’s results do not refute our earlier results, but instead, add important context that we hope will improve the field’s understanding of replicability. In light of Nee’s report, we reiterate a point that may have been under-appreciated from our prior report. The central idea is that task-based fMRI replicability depends on a multitude of factors that extend beyond sample size and individual-level data. Here, we present further evidence to support this conclusion.

Unfortunately, a direct comparison of the results from our earlier report and Nee’s new findings is limited by methodological differences between our two efforts. For example, Nee applied an alternative measure of cluster-level overlap, constructs his group maps using voxelwise one-sample *t*-tests rather than the mixed-effects approach used in our study, and uses one-tailed thresholding for his threshold-based replicability measures. All of our choices were the result of careful consideration, but we are sure Nee similarly had reasons behind making the changes he did; nonetheless, they hinder direct comparison between our results and Nee’s, at least for some measures.

That caveat notwithstanding, Nee convincingly demonstrated that in his cognitive control task, some contrasts are capable of attaining high levels of replicability at relatively low sample sizes, provided sufficient individual-level sampling. However, the relative weight of evidence suggests that we should not conclude that sample size is irrelevant, even with high degrees of individual-level sampling: Nee’s results are based on an analysis of a single task with a sample size of 46 (all of which are included in his tests of *N* = 23, which artificially shrinks his confidence intervals; see Supplementary Tables 2 and 3), whereas our results are based on an analysis of eleven tasks with a combined sample size of 956 unique individuals. Thus, the generalizability of Nee’s results await confirmation, as they may reflect characteristics of this specific experimental paradigm—for example, although some of Nee’s contrasts evince high replicability with 4–6 runs, those same contrasts are at least as replicable as our most replicable tasks, and by some measures much more so, even with only 1 run, suggesting that his paradigm differs from the 11 we examined previously in ways that cannot be explained by individual-level sampling.

Nee’s report also leaves unanswered the question of where individual-level sampling fits among the full set of variables that could impact replicability, because his analysis focuses on a single variable. Furthermore, there is still the question of which aspect of individual-level data is most important—total scanning duration, summated per-event duration, or (most likely) overall contrast power^3,4^. Indeed, the differences in replicability between his most-replicable and least-replicable contrasts, which were equivalent in terms of the first two of those variables, were larger than the differences within any contrast between 1 and 6 runs (a pattern which was also true in our report).

Taking these points together with the results of the additional analyses we conducted in our first study, we do not feel it is appropriate to conclude, as Nee does, that our results “cannot be broadly generalized to basic science research that tends to scan much longer”. Rather, more work remains to be done to delineate how sample size and individual-level sampling jointly contribute to replicability. To this end, and in order to illustrate the point that replicability is determined by a multitude of factors, we present results from two new analyses: first, we used Nee’s publicly available code to exactly replicate his analyses using another previously published dataset^5^, and second, we carried out an investigation of the relationships amongst a subset of the variables from the Measurables analysis of our published study^1^, using both the results of Nee and the new analyses carried out here. To preview, both of these analyses provide evidence that neither sample size nor individual-level sampling alone are sufficient to explain replicability.

The data in this task (for which participants provided informed consent; all procedures approved by UCSB’s IRB), which was designed to examine how target probability affected decision-making in recognition memory, were collected as a single 22.5-min-long functional run, which was split into six pseudo-runs of 60 trials each (fewer than Nee’s 160 trials/run, but at 1.5 s/trial versus Nee’s of 0.5 s, comparable in terms of total duration). We considered five orthogonal contrasts, each comprising ~30 trials per event per pseudo-run. Figure 1 below is analogous to Fig. 2 from Nee (see also Supplementary Figures 1–3). Replicability is much worse, and improves only marginally as runs are added.

**Fig. 1:**
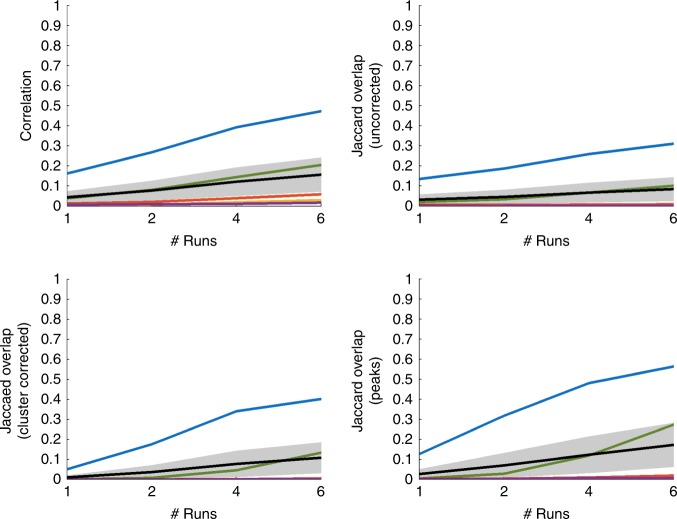
Replicability estimates at *N* = 23. Metrics correspond to those used in Nee, conservative thresholds

**Fig. 2:**
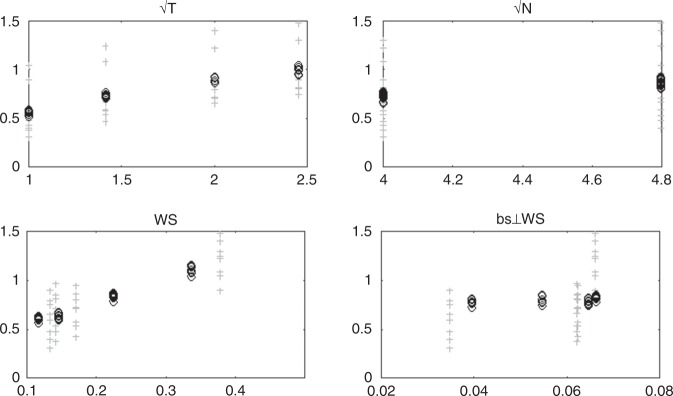
Scatterplots showing replicability (unthresholded, Fisher-transformed) as a function of the square root of the number of trials (*T*), the square root of the number of participants (*N*), the mean within-participant similarity across runs (ws), and the mean between-participant similarity marginalized w.r.t. within-participant similarity (bs⊥ws). Gray + show the raw values of all variables, while black ○ show the residuals (centered on the original mean) of each variable w.r.t. the remaining three variables

The second analysis attempted to relate replicability to several possible explanatory variables, including: (1) sample size; (2) number of trials; (3) within-participant variability (average whole-brain similarity between adjacent runs within a participant); and (4) between-participant variability (average pairwise whole-brain similarity between participant-level maps for all pairs). We correlated the Fisher-transformed mean estimate of unthresholded replicability (for each sample size, number of trials, and contrast) with each of the four above-mentioned explanatory variables, yielding a set of four correlations across 32 observations (Nee’s 4 contrasts × 2 levels of *N* × 4 levels of *T*). The results are presented in Fig. 2 and Supplementary Table 1 (see also Supplementary Figures 4 and 5), and demonstrate that whether measured by correlation or partial correlation, sample size exhibits a strong relationship despite a severely truncated range, and that within-participant similarity surpasses number of runs in the strength of its relationship with replicability.

Considering the results presented here alongside those of our previous work^1^ and Nee^2^, we conclude that: (1) sample size and individual-level sampling both impact replicability, alongside a host of other variables; (2) sample size is likely to play a strong role; and (3) although per-participant data surely plays a role, we disagree that this variable should be taken to invalidate our published results, or that it is a driver to the same extent as some other variables. Nonetheless, we are encouraged by this exchange, which is the sort of exploration we hoped our initial paper might catalyze. Replicability depends upon a multitude of factors which are difficult for any single analysis to measure. Indeed, the central aim of this effort is to motivate a field-wide change in the methodological conventions we employ, so that it becomes standard for researchers to run these analyses and report variables including replicability, as well as within-participant and between-participant variability.

## Supplementary information

Supplemental Material

## Data Availability

The datasets analyzed during the current study are not publicly available due to institutional restrictions on the sharing of confidential individual-level data, but individual and group SPMs are available from the corresponding author upon request.
