# Supplementary material for: Reply to: fMRI replicability depends upon sufficient individual-level data
Source: Commun Biol. 2019 Apr 12;2:129. doi: 10.1038/s42003-019-0379-5 (PMC6461603; doi:10.1038/s42003-019-0379-5)
Supplement: Supplementary file 1 — Supplemental Material [file 42003_2019_379_MOESM1_ESM.docx]

**Supplementary Figures**


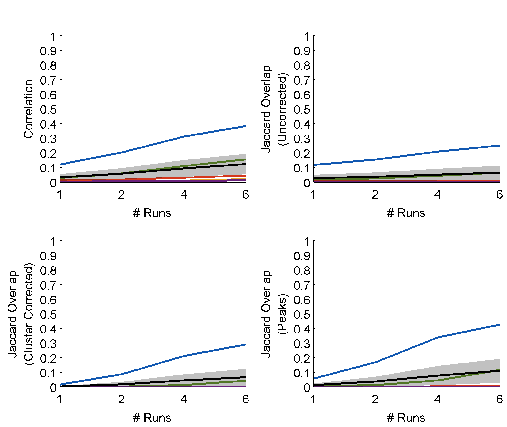


**Supplementary Figure 1**. Replicability estimates at N=16. Metrics correspond to those used in Nee, conservative thresholds.


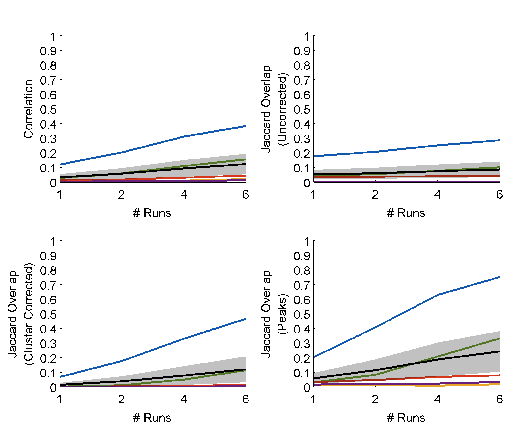


**Supplementary Figure 2**. Replicability estimates at N=16. Metrics correspond to those used in Nee, liberal thresholds.


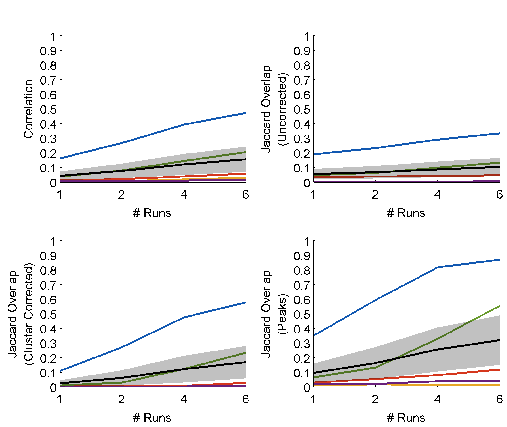


**Supplementary Figure 3**. Replicability estimates at N=23. Metrics correspond to those used in Nee, liberal thresholds.


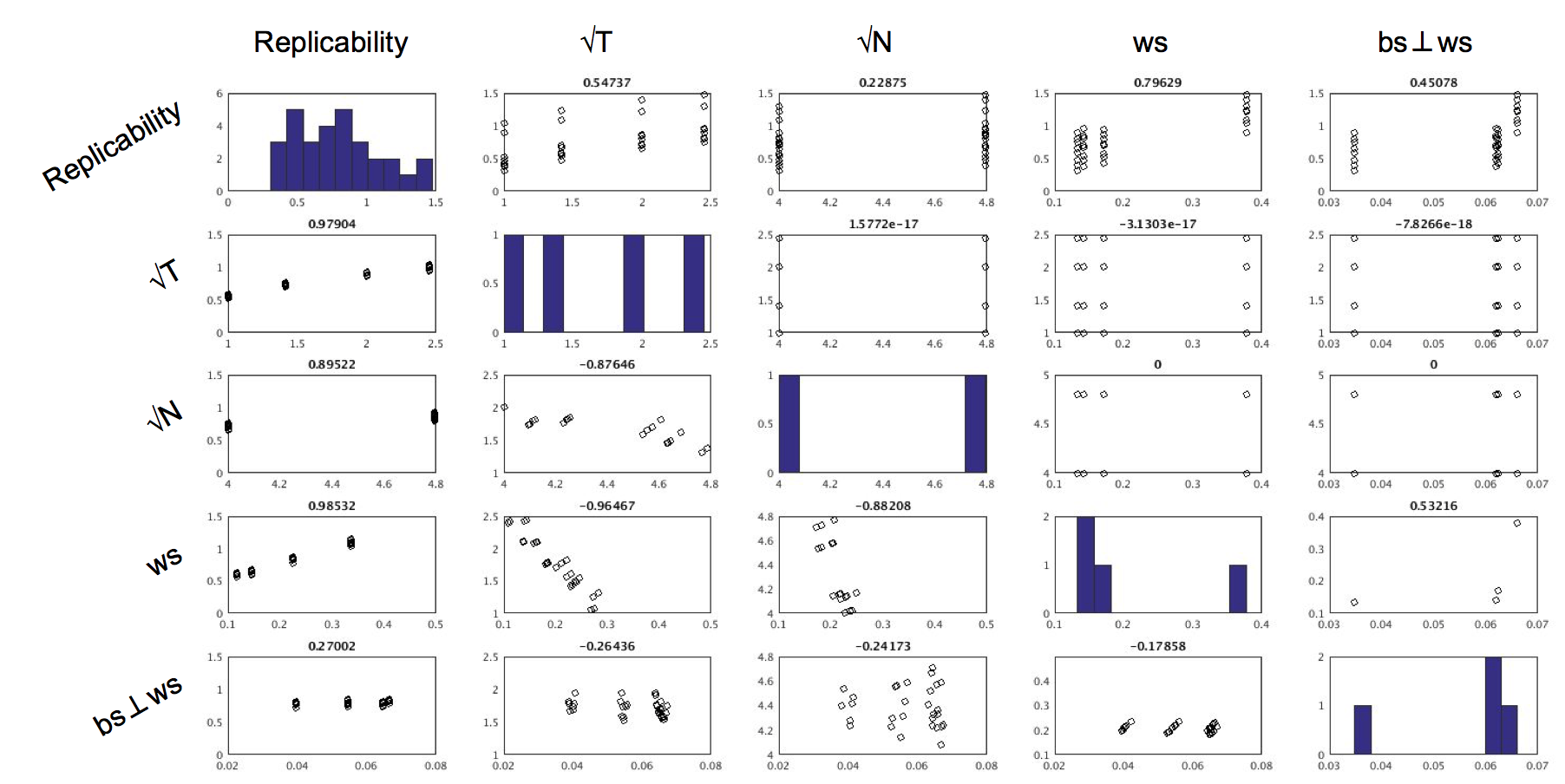


**Supplementary Figure 4.** Hybrid scatterplot matrix showing (above the diagonal) the relationship between each pair of raw variables, as well as (below the diagonal) the relationship between each pair of variables residualized w.r.t. the other three, with histograms of the raw variables on the diagonal. Shows data only for Nee’s task. Subplot titles give the correlation value corresponding to the plotted variables. √T: square root of the number of functional runs; √N: square root of the number of participants; ws: mean within-participant similarity; bs⊥ws: mean between-participant similarity orthogonalized with respect to mean within-participant similarity.


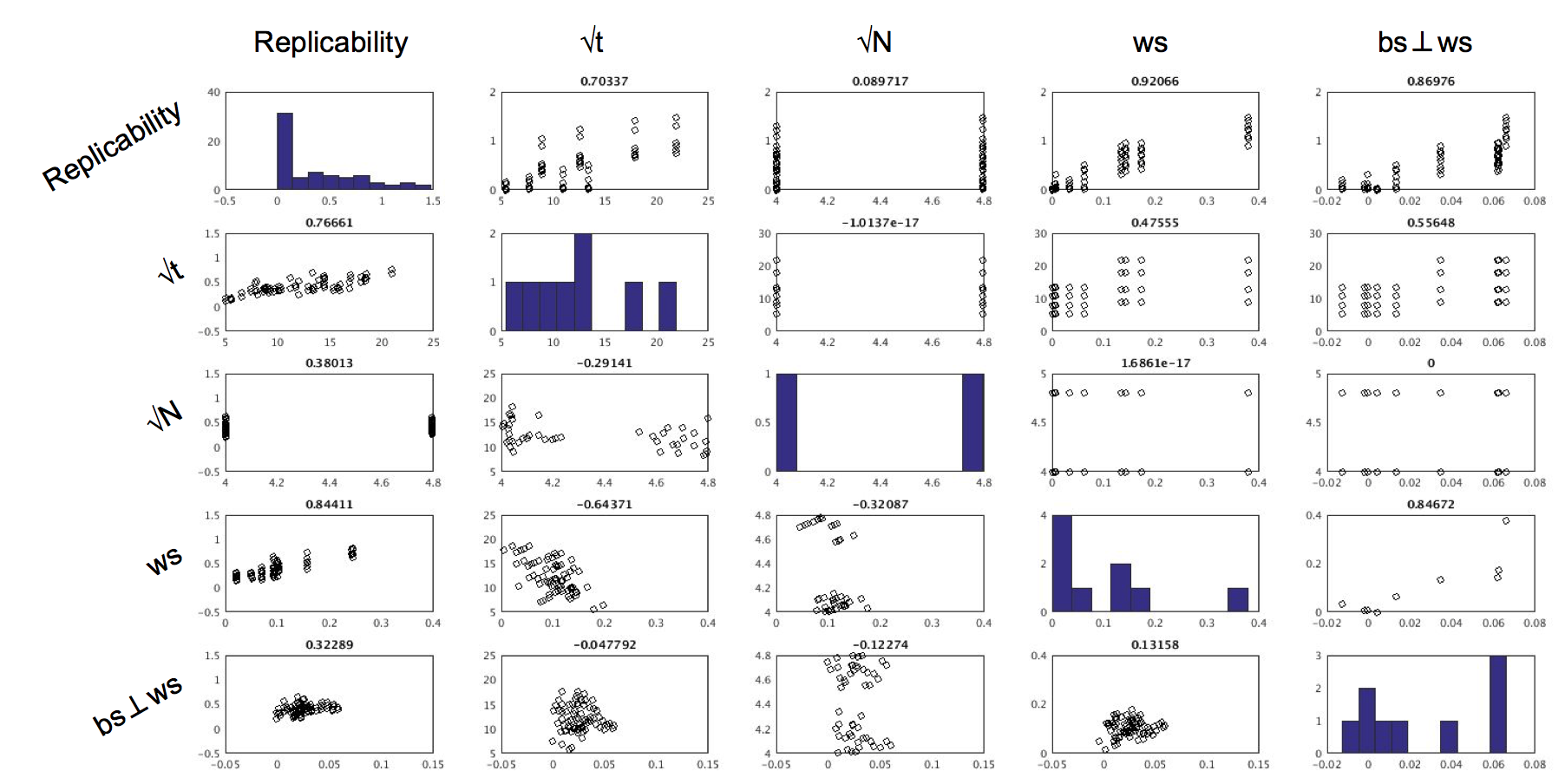


**Supplementary Figure 5.** Hybrid scatterplot matrix showing (above the diagonal) the relationship between each pair of raw variables, as well as (below the diagonal) the relationship between each pair of variables residualized w.r.t. the other three, with histograms of the raw variables on the diagonal. Shows combined data for Nee’s task as well as the task used in the other analyses presented here. Subplot titles give the correlation value corresponding to the plotted variables. √t: square root of the number of trials; √N: square root of the number of participants; ws: mean within-participant similarity; bs⊥ws: mean between-participant similarity orthogonalized with respect to mean within-participant similarity.

**Supplementary Tables**

**Supplementary Table 1.** Correlations and partial correlations between each of four explanatory variables and unthresholded replicability, using Nee’s results. We do not present confidence intervals because the values that were included in the correlations were not independent, so these are meant only as descriptive measurements of the specific relationships evident in Nee’s results.

|  | **√T** | **√N** | **ws** | **bs⊥ws** |
| --- | --- | --- | --- | --- |
| **Correlation** | 0.55 | 0.23 | 0.80 | 0.45 |
| **Partial correlation** | 0.98 | 0.90 | 0.99 | 0.27 |

**Supplementary Table 2.** Ratio of the standard deviation across 500 iterations using a subset of 46 participants over the standard deviation across 500 iterations using the full sample of 95 participants. Numbers in parentheses indicate for how many contrasts (out of 5) this ratio included a 0 in neither the numerator nor denominator.

| # runs | measure | **R** | **JvoxCon** | **JclustCon** | **JpeakCon** |
| --- | --- | --- | --- | --- | --- |
| **1** | | 0.9831 (5) | 1.1161 (5) | 1.0939 (2) | 0.8448 (4) |
| **2** | | 0.9351 (5) | 1.0487 (5) | 0.7770 (3) | 0.9847 (4) |
| **4** | | 0.7874 (5) | 0.8441 (5) | 0.5463 (4) | 0.6507 (4) |
| **6** | | 0.6013 (5) | 0.3618 (4) | 0.7212 (2) | 0.5227 (4) |

**Supplementary Table 3.** Difference between the average absolute difference of paired and unpaired iterations, expressed as a proportion of the unpaired standard deviation (see text for further explanation). Numbers in parentheses indicate for how many contrasts (out of 5) this proportion included a 0 in neither the numerator nor denominator.

| # runs | measure | **R** | **JvoxCon** | **JclustCon** | **JpeakCon** |
| --- | --- | --- | --- | --- | --- |
| **1** | | 0.9911 (5) | 0.9435 (5) | 0.9996 (2) | 1.0292 (4) |
| **2** | | 0.9630 (5) | 0.9513 (5) | 0.9503 (3) | 0.9650 (4) |
| **4** | | 0.8977 (5) | 0.9295 (5) | 1.0006 (4) | 0.9933 (5) |
| **6** | | 0.8730 (5) | 0.8449 (5) | 0.9475 (4) | 0.8945 (4) |

**Supplementary Methods**

We conducted a third set of analyses aimed to quantify the extent to which having zero degrees of freedom affected the generalizability of Nee’s results. We measured this effect in two ways: first, we compared the above analysis, which drew pseudo-replicate samples from the full sample of 95 participants for each iteration, to the results from an analysis that used a random sub-sample of 46 (that is, simulating Nee’s situation and using only these 46 throughout). In particular, we took the ratio of the sub-sample standard deviation over the full-sample standard deviation for each measure of replicability for the case of N=23. The results are presented in Supplementary Table 2. The second way we measured this effect was to constrain the sub-sample from which we drew participants on every other iteration. In particular, for iteration 1 (and separately for 3, …, 499), we drew 46 participants from the full set of 95 (and split them into two groups of 23). For iteration 2 (, 4, …, 500) we drew the 46 participants from the remaining 49 that weren’t included in iteration 1 (, 3, …). For each iteration, we otherwise carried out the analysis using Nee’s code. In this case, the measure of interest is how the absolute difference in replicability between paired iterations compares against the absolute difference in replicability between unpaired iterations. We express this difference in units of standard deviation across the 250 odd iterations—i.e., this is a signed difference of two absolute differences, expressed as a proportion of a standard deviation. The results of this analysis are presented in Supplementary Table 3.
